# Self-reported data validity for assessment of systemic and oral health as risk for dependency in old age: a cohort profile of elderly individuals in mid Sweden

**DOI:** 10.3389/froh.2025.1491723

**Published:** 2025-03-06

**Authors:** Alessandra Neves-Guimaraes, Ruzan Udumyan, Kartheyaene Jayaprakash Demirel, Pernilla Larsson Gran, Carin Starkhammar, Carina Källestål

**Affiliations:** ^1^ Department of Odontological Research, Public Dental Service, Faculty of Medicine and Health, Örebro University, Örebro, Sweden; ^2^ Department of Periodontology and Implantology, Public Dental Service, Faculty of Medicine and Health, Örebro University, Örebro, Sweden; ^3^ Clinical Epidemiology and Biostatistics, School of Medical Sciences, Örebro University, Örebro, Sweden; ^4^ Department of Oral and Maxillofacial Surgery, Faculty of Medicine and Health, Örebro University, Örebro, Sweden; ^5^ Department of Prosthodontics, Faculty of Odontology, Malmö University, Malmö, Sweden; ^6^ Centre of Oral Rehabilitation, Folktandvården Östergötland, Norrköping, Sweden; ^7^ Center for Oral Rehabilitation, Linköping, and Department of Biomedical and Clinical Sciences, Linköping University, Linköping, Sweden; ^8^ Department of Dental Research, Public Dental Service, Region Örebro County, Faculty of Medicine and Health, Örebro University, Örebro, Sweden

**Keywords:** aging, oral health, general health, dependency, longitudinal, selection bias

## Abstract

**Purpose:**

The Mid Sweden Cohort (MSC) was established to investigate self-perceived oral and general health among two groups of aging individuals in two counties (Örebro and Östergötland) in Sweden. For internal and external data validation, we linked collected data on health status, behavior, sociodemographic circumstances, and dependency with national register data from Statistics Sweden and compared non-respondents and those lost to follow-up to respondents.

**Participants:**

MSC is based on a longitudinal multiwave study of aging men and women who answered a cross-sectional questionnaire from MSC: (1) the 1992 cohort including participants aged 50 years in 1992 and (2) the 2007 cohort including participants aged 75 years in 2007. After the baseline surveys, data collection was conducted every 5 years, with the latest wave from 2017 included in our validation. Between 1992 and 2017, 8,879 participants were included in cohort 1, while 5,191 individuals were included in cohort 2 between 2007 and 2017.

**Results:**

After linking self-reported data with national register-based data and analyzing loss to follow-up and non-response numbers, we found that, besides age, factors such as being male, having immigrant status, lower income and education level, being single, and being in poor health were predictors of non-response and loss to follow-up, aligning with the findings of other studies. Based on our results, we conclude the MSC is reliable for further research, provided the observed bias is taken into account.

**Future plans:**

Using the MSC, we aim to analyze self-reported oral health changes as a predictor of dependency in the elderly and track oral health status over time. Furthermore, we plan to link data with register-based clinical oral health records. We also intend to add the 2022 wave data and future waves into the existing dataset.

## Introduction

According to the World Health Organization (WHO), the number of people aged over 60 years will double by 2050, comprising approximately 22% of the global population and necessitating appropriate healthcare strategies to allow for healthy aging (1, 2).

Aging-related immunological, physical, and cognitive changes predispose individuals to a range of oral and systemic chronic inflammatory diseases (3, 4).

Oral diseases are one of the most prevalent conditions worldwide. Due to demographic changes especially related to population growth and aging, the cumulative burden of oral conditions (e.g., untreated dental caries or tooth loss) increased dramatically between 1990 and 2015, from 2.5 to 3.5 billion untreated people (5, 6). Unfortunately, data on the oral status of elders are generally sparse (7).

The association between oral and general health has gained increasing interest in recent years. Although studies focusing on the link between oral health status and general health, dependency, or frailty among the elderly are scarce, some have investigated the association between the deterioration in oral status and frailty (8–10). A longitudinal study from Japan found that poor oral health is a strong predictor for the onset of adverse health outcomes, including mortality among community-dwelling elderly individuals (3).

From a methodological perspective, many published studies in this area are, at least in part, based on self-reported data. The prime advantage in using self-reported data is the possibility to assess individuals’ own experience and perceptions in relation to their health. On the other hand, some intrinsic limitations are the risk of bias due to social desirability or approval and recall bias (11, 12). Many aging prospective cohorts are underway globally, designed to follow the natural course of events, assess changes over time, and catch temporality in studied associations (13).

The aim of the present study was to present and assess the potential threats to validity of self-reported data from two elderly cohorts in two counties in mid Sweden (Örebro and Östergötland regions), known as the Mid Sweden Cohort (MSC). Validation of data through clinical examinations conducted during the first data collection in 1992 for one of the cohorts showed a good correspondence between subjective self-reports and clinical findings, particularly for conditions that are relatively easy for patients to observe, such as the number of permanent teeth or the presence of dentures (14).

Our specific objectives were

- to present the cohorts with a focus on methodological design and available variables,
- to evaluate the agreement between self-reported and register-based data (obtained from Statistics Sweden—SCB) on socioeconomic variables (internal validity), and
- to compare respondents with non-respondents and those lost to follow-up (external validity).

## Methods

### Study population and data collection

The current and future studies will be based on data from two MSC cohorts: (1) a cohort including participants aged 50 years in 1992 (referred to as the 1992 cohort) and (2) a cohort including participants aged 75 years in 2007 (referred to as the 2007 cohort).

Data collection has been previously described (14). Briefly, the addresses of potential participants were obtained from public population records (Statistics Sweden—SCB). Individuals were excluded if a questionnaire was returned with an unknown address. If individuals did not respond within 2 weeks, a reminder was sent in the form of a letter. A new questionnaire was mailed to those who did not answer after these additional 2 weeks. No further contact was attempted thereafter.

**Figure 1:**
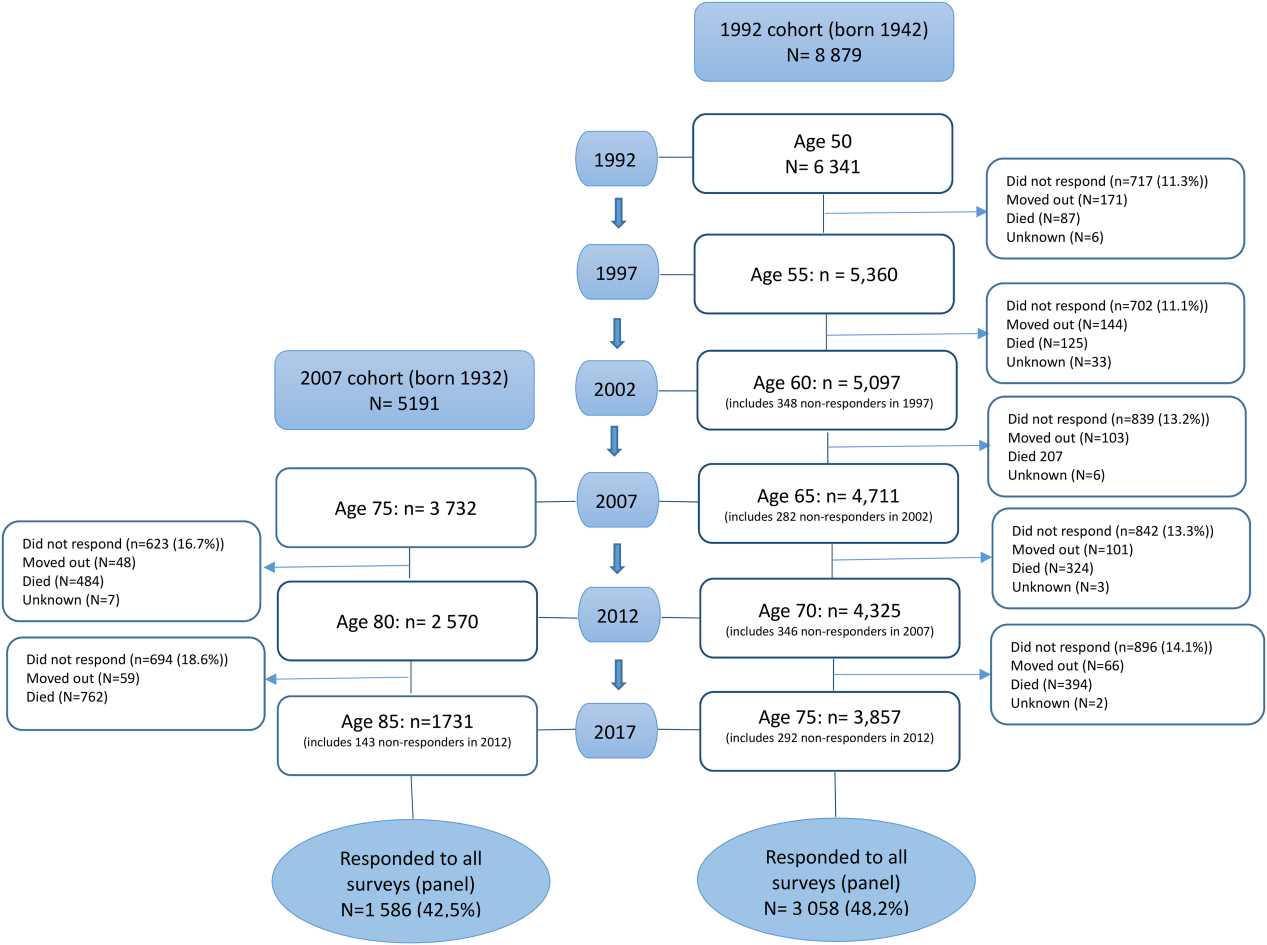
Responders during each data collection wave among respondents to the baseline survey.

After the baseline surveys, data collection was conducted every 5 years, with the latest data collection from 2017 included in our validation. During follow-up surveys, the questionnaire was sent to all individuals born in 1942 and 1932 living in the two counties. The cohorts are open: individuals contacted during the follow-up surveys are not only those who have been approached for the baseline surveys. For example, people who moved to these counties after the baseline surveys were also eligible. Figure 1 shows a flowchart depicting the data collection process for the 1992 and 2007 cohorts among respondents to the baseline surveys.

### Self-reported questionnaire data

The questionnaire stayed largely unchanged throughout the data collection period to ensure the comparability of data (15). The questionnaire design has been described previously (15). All variables with their original coding are presented in Supplementary Table S1.

The questionnaire includes questions on

• general sociodemographic conditions (age, sex, place of birth, residential area, education, marital status),
• lifestyle factors (smoking habits and alcohol consumption),
• anthropometric measurements (weight and height used to derive body mass index),
• oral and general health (satisfaction with general health, oral health, toothache, number of teeth, problems in chewing, burning mouth), and
• dependency (assessed only in the survey conducted in 2017).

For the current analyses, we used the variable of sex, as well as dichotomized or recoded categorical variables, such as country of birth (“born in Sweden,” “born abroad”), education (“less than university,” “university”), marital status (“married/cohabiting,” “unmarried/divorced/widowed”), smoking habits (“daily,” “less than daily”), tooth brushing (“twice a day or more,” “less than twice a day”), perceived oral health (“very/largely satisfied” or “not very/absolutely not satisfied”), and perceived general health (“healthy,” “not healthy,” “have no opinion”).

### National register-based data

Population-based registers held by Statistics Sweden provided information on background variables (sex, year of birth, country of birth [recoded as “Sweden,” “foreign country”]), migration, and vital status, as well as variables included in the Longitudinal Database of Education, Income and Occupation (Swedish acronym: LISA). From LISA, we used level of attained education (recoded as “compulsory,” “secondary,” “post-secondary”), disposable income (created tertiles 1 = lowest, 3 = highest), marital status (recoded as “unmarried,” “married/cohabiting,” “divorced/separated,” “widower”), and county and municipality of residence (used to derive degree of urbanization variable) (16) for the years that correspond to survey years. For the years 2012 and 2017, we also obtained information on the form regarding accommodation (apartment, small house, special housing, or other accommodation) but did not use the data in the current analysis.

Survey data were linked to the national registry data using the unique personal identification number assigned to all Swedish residents. Data from national registers were obtained for both survey respondents and non-respondents.

### Statistical methods

We compared the distribution of sociodemographic variables obtained from national registers between participants and non-respondents to baseline surveys in 1992 and 2007 using the chi-square test (Table 2). To identify the predictors for non-response to the baseline survey, we performed univariate and multivariable logistic regression analyses for all eligible participants (Table 3) and among men and women separately, although the multiplicative interaction term with sex was non-significant (Supplementary Table S3). All sociodemographic variables (sex, country of birth, degree of urbanization of the residential area**,** level of attained education, marital status, disposable income, and county of residence) were included in the analyses.

Predictors for loss to follow-up were investigated in individuals who participated in the baseline surveys. The response to each follow-up survey was analyzed separately, since participants could be temporarily lost to follow-up. Participants who were lost to follow-up due to moving to other counties, emigration, or death were excluded from these analyses. The covariates were from the baseline survey period (Supplementary Tables S4a,b) or from a preceding survey period (Supplementary Table S5). In addition to the sociodemographic variables obtained from national registers, the logistic regression models included health-related (perceived oral health and perceived general health) and behavioral (smoking and tooth brushing habits) variables obtained from the survey questionnaires. Missing values were included as a separate category for variables from follow-up surveys (smoking, tooth brushing habits, and perceived oral and general health).

In further analyses, we compared individuals who participated in all assessments (panel) and would have been included in the complete-case analysis with those who did not respond at some point after the baseline surveys (Table 4). In this analysis, dropout also includes individuals who were lost to follow-up due to leaving the study counties or death. Cox regression was used to study the association between baseline covariates and mortality among the respondents to baseline surveys.

Collinearity diagnostics, performed using pairwise correlations and the variance inflation factor (VIF) (17), did not detect collinearity between covariates. Goodness of fit of the logistic regression models was examined using the Hosmer–Lemeshow test.

We also assessed the agreement [i.e., concordance between the two sets of measurements (18)] between sociodemographic variables reported by the survey respondents and obtained from the national registers. We calculated the percent agreement (i.e. the observed proportion of agreement) between the two sets of measurements as the number of individuals whose response to survey was confirmed in the registers divided by the total number of respondents to baseline surveys (19) (Table 5). We also reported chance-corrected agreement coefficients (ACs), such as frequently used Cohen's kappa statistic and Gwet's AC (19). To interpret the results, the benchmarking scale proposed by Landis and Koch was used (19, 20). It classifies values <0 as indicating poor agreement, 0–0.20 as slight agreement, 0.21–0.40 as fair agreement, 0.41–0.60 as moderate agreement, 0.61–0.80 as substantial agreement, and 0.81–1 as almost perfect agreement (19, 20). The cumulative probability for a coefficient to fall into the benchmark interval (using the probabilistic benchmarking method) was also estimated (19).

Stata version 17/MP (StataCorp, College Station, TX, USA) was used for statistical analyses (21).

### Ethics and dissemination

The original studies from 1992 to 2007 were approved by the Ethics Committee in Sweden. Because of new regulations, approval of the follow-up questionnaires by an ethics committee was not required until 2012 (Dnr2006/251). Due to modifications of the questionnaire in 2017, new approval was obtained from the same year for both cohorts (Dnr 2016/424). Additional ethical approval was obtained in 2021 and completed in 2022 (Dnr 2021-02353 and Dnr 2022-04247-01) to enable the linking to the register-based data by Statistics Sweden and the Swedish National Board of Health and Welfare for validity checks and further analyses.

Neither the participants nor the general public were involved in the planning or design, recruitment, or conduction of the study.

## Results

### Study population and response

In 1992, a questionnaire was sent to all individuals aged 50 years (*N* = 8,888) in two counties in Mid Sweden: Örebro and Östergötland. For the current analysis, we excluded individuals with reused personal numbers (*n* = 9; in Sweden, social identification numbers can be reused from deceased individuals to immigrants and thus is no longer unique to an individual and information linked to the social security number refers to a different person). Of the remaining 8,879 individuals, 6,341 (71.4%) responded to the baseline survey in 1992, of whom 3,058 (48.2%) also participated in all subsequent data collection waves in 1997, 2002, 2007, 2012, and 2017 (53.4% women and 46.6% men) and comprised the 1992 panel data (Table 1, Supplementary Table S2, Figure 1). The overall response rate to each survey varied between 70.7% and 75.0% (Supplementary Table S2) and was in the range of 71.3%–75.6% among those who were eligible for the baseline survey (Table 1).

**Table 1:** Response rates across survey waves among eligible individuals in both cohorts.

Cohorts	Survey year	Age, years	Female responders (%)	Male responders (%)	Total responders (%)	Total (% of response at baseline)
1992 cohort	1992	50	3,181 (50.2)	3,160 (49.8)	6,341 (71.4)	
	1997	55	3,215 (50.9)	3,104 (49.1)	6,319 (74.9)	
	2002	60	3,102 (51.1)	2,964 (48.9)	6,066 (75.6)	
	2007	65	2,858 (50.6)	2,786 (49.4)	5,644 (73.6)	
	2012	70	2,624 (50.7)	2,549 (49.3)	5,173 (72.8)	
	2017	75	2,391 (52.2)	2,189 (47.8)	4,580 (71.3)	
	Cohort by survey year					
	92/97	50/55	2,751 (51.3)	2,609 (48.7)		5,360 (84.5)
	92/97/02	50/60/65	2,458 (51.9)	2,275 (48.1)		4,733 (74.6)
	92/97/02/07	50/55/60/65	2,162 (52.2)	1,979 (47.8)		4,141 (65.3)
	92/97/02/07/12	50/55/60/65/70	1,876 (52.4)	1,707 (47.6)		3,583 (56.5)
	92/97/02/07/12/17	50/55/60/65/70/75	1,633 (53.4)	1,425 (46.6)		3,058 (48.2)
2007 cohort	2007	75	1,987 (53.2)	1,745 (46.8)	3,732 (71.9)	
	2012	80	1,561 (54.5)	1,303 (45.5)	2,864 (66.9)	
	2017	85	1,089 (55.9)	859 (44.1)	1,948 (61.7)	
	Cohort by survey year					
	07/12	75/80	1,389 (54.1)	1,181 (46.0)		2,570 (68.9)
	07/12/17	75/80/85	859 (54.2)	727 (45.8)		1,586 (42.5)

In the 2007 cohort of 5,191 eligible individuals aged 75 years, 3,732 (71.9%) responded to the baseline survey in 2007 (53.2% women and 46.8% men). A total of 1,586 (42.5%) individuals completed the questionnaires at 75, 80, and 85 years of age (54.2% women and 45.8% men), comprising the 2007 panel data (Table 1, Figure 1). The overall response rate to each survey varied between 61.6% and 71.9% (Supplementary Table S2) and was in the range of 61.7%–71.9% among those who were eligible for the baseline survey (Table 1).

The response rates were similar in the two counties (15). Among the respondents to the baseline survey, non-response to subsequent surveys was in the range of 11.1%–14.1% in the 1992 cohort and 16.7%–18.6% in the 2007 cohort (Figure 1). The dropout from subsequent surveys was partly due to a move to a different county/country or death (Figure 1).

### Factors associated with non-response at baseline

In the 1992 cohort, the majority of non-respondents were men, while in the 2007 cohort the majority of non-respondents were women (Table 2). In both cohorts, non-respondents were more likely to be born abroad, have a lower degree of attained education, be single (unmarried or divorced/separated or widowed), and have a lower disposable income (Table 2). Missing data for these variables (from national registers) was in the range of 0.0%–1.8%, and the highest proportions of missing values were observed for the attained education variable (1.8% in the 2007 cohort) and the residential area by degree of urbanization variable (0.51% in the 1992 cohort and 0.52% in the 2007 cohort).

**Table 2:** Distribution of sociodemographic characteristics of responders and non-responders to the baseline surveys (in 1992 and 2007) in both cohorts (1992 and 2007) using data from national registries.

Sociodemographic characteristics from National Registers	1992 cohort (born in 1942) *N* = 8,879			2007 cohort (born in 1932) *N* = 5,191		
	Respondents (*N* = 6,341)	Non-respondents (*N* = 2,538)	*p*-value	Respondents (*N* = 3,732)	Non-respondents (*N* = 1,459)	*p*-value
Age, years	50	50		75	75	
Men, %	49.8	53.7	0.001	46.8	41.3	<0.001
Place of birth, %			<0.001			<0.001
Sweden	93.5	90.0		92.0	83.4	
Foreign country	6.5	10.0		8.0	16.6	
Residential area by degree of urbanization, %			0.093			0.300
Degree 1 (cities)	54.5	52.7		51.0	50.3	
Degree 2 (towns and suburbs)	23.7	24.0		25.2	23.8	
Degree 3 (rural areas)	21.4	22.6		23.3	25.2	
Missing	0.4	0.7		0.5	0.7	
Education, %			<0.001			<0.001
Compulsory	34.8	46.2		47.8	60.5	
Secondary	40.6	37.6		34.8	27.4	
Post-secondary	24.5	15.3		16.5	7.7	
Missing	0.2	0.9		0.8	4.4	
Marital status, %			<0.001			<0.001
Unmarried	10.6	15.4		4.9	8.8	
Married/cohabiting	73.0	62.3		62.1	50.7	
Divorced/separated	14.6	20.1		12.4	15.3	
Widower	1.8	2.1		20.6	25.2	
Missing	0.0	0.0		0.0	0.0	
Tertiles of disposable income, %			<0.001			<0.001
1 (low)	30.6	40.3		30.5	40.8	
2 (medium)	33.6	32.6		32.4	35.7	
3 (high)	35.8	27.1		37.1	23.5	
Missing	0.0	0.0		0.0	0.0	

*p*-values are from chi-squared test.

In univariate and multivariable analyses of predictors of non-response to the baseline survey in 1992, male sex, foreign country of birth, and being single showed positive associations, while higher degrees of education and disposable income showed inverse associations with non-response (Table 3). Similar patterns were observed in the 2007 cohort. However, male sex was inversely associated with non-response in the crude analysis and showed a near-null association in the adjusted analysis (Table 3). Degree of urbanization of residential area and counties (Örebro vs. Östergötland) were not associated with non-response to the baseline surveys. Associations of sociodemographic variables with non-response were largely similar among men and women (Supplementary Table S3).

**Table 3:** Univariate and multivariable logistic regression analyses of predictors for non-response to the baseline surveys (in 1992 and 2007) in both cohorts (1992 and 2007) using baseline data from national registries—complete-case analysis.

Sociodemographic characteristics from national registers	1992 cohort (born in 1942) *N* = 8,802		2007 cohort (born in 1932) *N* = 5,070	
	cOR (95% CI)	aOR (95% CI)	cOR (95% CI)	aOR (95% CI)
Men	1.17 (1.06–1.28)	1.35 (1.21–1.50)	0.81 (0.71–0.91)	1.06 (0.91–1.23)
Country of birth				
Sweden	Ref.	Ref.	Ref.	Ref.
Foreign country	1.56 (1.32–1.84)	1.45 (1.22–1.72)	1.97 (1.62–2.41)	1.90 (1.55–2.34)
Residential area by degree of urbanization				
Degree 1 (cities)	Ref.	Ref.	Ref.	Ref.
Degree 2 (towns and suburbs)	1.06 (0.95–1.19)	1.02 (0.91–1.15)	0.99 (0.85–1.15)	0.92 (0.78–1.07)
Degree 3 (rural areas)	1.10 (0.98–1.24)	1.03 (0.92–1.17)	1.13 (0.97–1.31)	1.06 (0.90–1.24)
Education				
Compulsory	Ref.	Ref.	Ref.	Ref.
Secondary	0.70 (0.63–0.77)	0.74 (0.67–0.83)	0.62 (0.54–0.72)	0.67 (0.58–0.77)
Post-secondary	0.47 (0.41–0.53)	0.58 (0.50–0.67)	0.37 (0.30–0.46)	0.50 (0.40–0.64)
Marital status				
Unmarried	1.69 (1.47–1.94)	1.54 (1.34–1.77)	2.28 (1.79–2.92)	2.30 (1.79–2.95)
Married/cohabiting	Ref.	Ref.	Ref.	Ref.
Divorced/separated	1.60 (1.41–1.81)	1.61 (1.42–1.83)	1.62 (1.35–1.94)	1.70 (1.40–2.05)
Widower	1.36 (0.97–1.89)	1.60 (1.14–2.26)	1.56 (1.34–1.82)	1.63 (1.37–1.93)
Tertiles of disposable income				
1 (low)	Ref.	Ref.	Ref.	Ref.
2 (medium)	0.74 (0.66–0.83)	0.69 (0.61–0.77)	0.87 (0.75–1.00)	0.76 (0.65–0.90)
3 (high)	0.58 (0.51–0.65)	0.59 (0.52–0.68)	0.50 (0.43–0.59)	0.55 (0.46–0.67)
County				
Östergötlands län	Ref.	Ref.	Ref.	Ref.
Örebro län	1.02 (0.93–1.12)	1.00 (0.91–1.10)	1.08 (0.96–1.23)	1.05 (0.92–1.19)

cOR, crude odds ratio; aOR, adjusted odds ratio; CI, confidence interval.

Missing values ranged from 0.0% to 0.5% in 1992 cohort and from 0.0% to 1.8% in 2007 cohort; and about 0.9% and 2.3% are excluded due to missing observations in 1992 and 2007 cohorts, respectively.

### Factors associated with dropout

Overall, in multivariable analyses using baseline predictors, being single, daily smoking, and being dissatisfied with oral and/or general health were associated with higher odds of dropout from subsequent surveys, while higher degrees of education and disposable income were associated with lower odds of dropout. For the 1992 cohort, male sex and birth in a foreign country also predicted dropout (Supplementary Tables S4a,b). These associations were statistically significant for most but not all follow-up surveys.

Largely similar patterns were observed in multivariable analyses using predictors from previous surveys (Supplementary Table S5).

A comparison of individuals who participated in all assessments and those who dropped out at some point after baseline surveys (Table 4) suggested that male sex, birth in a foreign country, lower level of education, being single, lower disposable income, daily smoking, brushing teeth less than twice a day, and being dissatisfied with oral and/or general health were statistically significantly associated with higher odds of dropout from subsequent surveys in the 1992 cohort. Largely similar patterns of associations were observed in the 2007 cohort. However, country of birth and oral health satisfaction did not reach statistical significance, and tooth brushing habits was marginally significant in the 2007 cohort.

**Table 4:** Logistic regression analysis results comparing individuals who participated in all assessments (panel) and those who dropped out at some point after the baseline surveys in 1992 and 2007.

Baseline covariates	1992 cohort *N*_A_ = 6,146	2007 cohort *N*_A_ = 3,203
Sociodemographic characteristics from National Registers	aOR (95% CI)	aOR (95% CI)
Male sex	1.52 (1.34–1.71)	1.29 (1.08–1.55)
Country of birth		
Sweden	Ref.	Ref.
Foreign country	1.67 (1.33–2.11)	1.26 (0.94–1.68)
Residential area by degree of urbanization		
Degree 1 (cities)	Ref.	Ref.
Degree 2 (towns and suburbs)	1.13 (1.00–1.29)	0.95 (0.80–1.13)
Degree 3 (rural areas)	1.05 (0.92–1.20)	1.14 (0.95–1.37)
Education		
Compulsory	Ref.	Ref.
Secondary	0.84 (0.74–0.94)	0.90 (0.77–1.06)
Post-secondary	0.95 (0.81–1.10)	0.68 (0.55–0.85)
Marital status		
Unmarried	1.78 (1.49–2.13)	1.42 (1.00–2.02)
Married/cohabiting	Ref.	Ref.
Divorced/separated	1.48 (1.27–1.72)	1.26 (0.99–1.59)
Widower	1.12 (0.76–1.66)	1.41 (1.16–1.73)
Tertiles of disposable income		
1 (low)	Ref.	Ref.
2 (medium)	0.80 (0.70–0.91)	0.73 (0.60–0.90)
3 (high)	0.63 (0.54–0.74)	0.68 (0.55–0.85)
County		
Östergötlands län	Ref.	Ref.
Örebro län	1.10 (0.99–1.23)	1.03 (0.88–1.19)
Information from baseline surveys		
Smoking		
Less than daily	Ref.	Ref.
Daily	1.86 (1.65–2.10)	1.98 (1.44–2.72)
Brushing		
Twice a day or more	Ref.	Ref.
Less than twice a day	1.16 (1.00–1.35)	1.20 (0.99–1.46)
Perceived oral health		
Very/largely satisfied	Ref.	Ref.
Not very/absolutely not satisfied	1.26 (1.10–1.44)	1.05 (0.87–1.26)
Perceived general health		
Healthy	Ref.	Ref.
Not healthy	1.98 (1.65–2.38)	2.19 (1.86–2.59)
Have no opinion	1.64 (0.77–3.47)	2.14 (1.01–4.55)

aOR, adjusted odds ratio; CI, confidence interval; *N*_A_, number in the adjusted model.

Observations with missing baseline values (3% for 1992 cohort and 14% for 2007 cohort) are excluded from the analysis [for baseline covariates, missing values ranged from 0.0% to 1.5% in 1992 cohort and from 0.0% to 9.5% in 2007 cohort (the 9.5% missingness was for tooth brushing habits)]. The dropout in this analysis includes participants who did not respond or died or moved out of the county or the country.

Among the respondents to the baseline surveys, 35% and 58% dropped out due to mortality in the 1992 and 2007 cohorts, respectively. Male sex, lower level of education, being single, daily smoking, and being dissatisfied with general health were statistically significantly associated with a higher mortality rate in both cohorts (data not shown). Lower disposable income was statistically significantly associated with a higher mortality rate in the 1992 cohort but not in the 2007 cohort.

### Agreement between self-reported and registry-based data at baseline

The percent agreement values indicate that the two sources agree on 92%–100% of the individuals (Table 5). Based on the Landis and Koch scale, the chance-corrected coefficients suggest substantial to almost perfect agreement for sex, country of birth, education, and marital status in both cohorts (Table 5). Using a probabilistic benchmarking approach, we can confirm this with 100% certainty. All coefficients were statistically significant.

**Table 5:** Agreement between baseline self-reported questionnaire and registry-based data on sociodemographic variables available in both sources.

Cohorts	Percent agreement	Cohen/Conger's kappa	Gwet's AC
	Coefficient (95% CI)	Coefficient (95% CI)	Coefficient (95% CI)
Cohort 1992			
Sex: male vs. female	1.00 (1.00–1.00)	1.00 (1.00–1.00)	1.00 (1.00–1.00)
Country of birth: Sweden vs. foreign country	1.00 (1.00–1.00)	0.99 (0.97–0.99)	1.00 (1.00–1.00)
Education: less than university vs. university	0.93 (0.92–0.94)	0.82 (0.80–0.83)	0.90 (0.88–0.91)
Marital status: married/cohabiting vs. unmarried divorced/widowed	0.90 (0.89–0.91)	0.72 (0.70–0.74)	0.85 (0.84–0.86)
Cohort 2007			
Sex: male vs. female	0.99 (0.98–1.00)	0.98 (0.97–0.99)	0.98 (0.97–0.99)
Country of birth: Sweden vs. foreign country	1.00 (0.99–1.00)	0.99 (0.98–1.00)	1.00 (0.99–1.00)
Education: less than university vs. university	0.92 (0.91–0.93)	0.68 (0.63–0.71)	0.90 (0.89–0.91)
Marital status: married/cohabiting vs. unmarried divorced/widowed	0.94 (0.93–0.95)	0.87 (0.85–0.88)	0.89 (0.87–0.90)

AC, agreement coefficient.

## Discussion

The validation of data is primordial for a critical understanding of selection bias and attrition bias and to ensure data quality in longitudinal aging research. Different approaches can be used for validation, including linking available data with register-based data (12).

The proportion participating in all the waves of the cohort study was over 70% for the 1992 cohort and over 60% for the 2007 cohort. According to a 2021 study by Sataloff and Vontela (22), these can be considered good response rates. Some characteristics predict subsequent non-response, e.g., is aging *per se* an issue (13). This is evident in the increasing proportion of participants leaving the cohort study over time, from 11.3% in the first wave of the 1992 cohort to 14.1% in the last wave in 2017. In the 2007 cohort, non-respondents increased from 16.7% to 18.6% by 2017.

Besides age, our results confirm findings from other studies indicating factors such as being a man, having immigrant status, lower income and education level, being single, and in poor health (23–25) as predictors of non-response. In Sweden, smoking is considered to be a social marker for lower social status; in our cohorts, smoking is associated with attrition, similar to other prospective studies (26).

At older ages, variables that predicted dropout at the beginning of the study, such as being male and having a low income, lose significance. One possible reason for this is that older individuals tend to have a more equal standard of living. For example, women and individuals from higher social strata generally have a longer life expectancy. It has also been reported that non-smoking women are more prevalent in older age groups because they live longer (27).

Self-reported data can be affected by social desirability or recall bias. However, we found substantial to almost perfect agreement between self-reported and register-based data. The lower kappa values observed, e.g., for education in the 2007 cohort, might be attributed to the so-called high agreement but low kappa paradox. The Gwet's AC results are more in line with the observed proportion of agreement and arguably represent the data more accurately.

The selection bias due to loss to follow-up with the more healthy, educated, and wealthy participating to a higher degree is expected and similar to most cohort studies. Based on the results of linking self-reported data to register-based data to assess selection bias, along with a meticulous statistical comparison of non-response and loss to follow-up, we conclude that the MSC is trustworthy for further studies. However, the selection bias shown in this validation must be taken into consideration when generalizing results to the broader population.

We plan studies using these data with the following overall research questions:

• Can we use self-reported oral health changes as predictors for future mortality and dependency in elderly?
• What are the self-reported oral health status changes in the two cohorts of elderly individuals?

We further consider linking clinical data to existing data to evaluate self-perceived oral health as a possible predictor for oral and general health as well as estimate periodontal and peri-implantits changes in aging people. We also hope to include the last wave of the cohort data collected in 2022 as well as future waves.

## Data Availability

The raw data supporting the conclusions of this article will be made available by the authors, without undue reservation.
