# Supplementary material for: Self-reported data validity for assessment of systemic and oral health as risk for dependency in old age: a cohort profile of elderly individuals in mid Sweden
Source: Front Oral Health. 2025 Mar 6;6:1491723. doi: 10.3389/froh.2025.1491723 (PMC11922726; doi:10.3389/froh.2025.1491723)
Supplement: Supplementary file 1 [file Table1.docx]

**Supplementary Table S1.** Variables obtained through questionnaires (self-reported). Highlighted with bold the variables used in this paper.

| **Question Variable** | | | Categories (original code) | **Recoded** categories |
| --- | --- | --- | --- | --- |
|  |  |  |  |  |
| *Sociodemographic factors* | Sex | **Sex** | 1 = Man  2 = Woman | 0 = Woman  1 = Man  9= Missing |
|  | Place of birth | **Place of birth** | 1 = Sweden  2 = Other Nordic country  3 = Other country | 0 = Born in Sweden  2-3 = 1 = Born abroad  9= Missing |
|  |  |  |  |  |
|  |  |  |  |  |
|  | Where do you live? | Residential area | 1 = Densely populated area  2 = Village  3 = Rural area | 0 = Village / Rural area  1 = Densely populated area  9= Missing |
|  | What education do you have? | **Education** | 1 = Elementary school  2 = Secondary school, 2-year high school or equivalent  3 = Three- or four-year high school  4 = University  5 = Other (open answer) | 0 = Less than university  1 = University  9 = Missing |
|  | What marital status do you have? | **Marital status** | 1 = Married/Cohabiting  2 = Unmarried  3 = Divorced  4 = Widowed  5 = Other (open answer) | 0 = Married/cohabiting  1 = Unmarried/divorced/widowed  9 = Missing |
| *Lifestyle factors* | Smoking habits | **Smoking** | 1 = Daily  2 = Stopped  3 = Occasionally  4 = Never smoked  9 = Wrong answer | 2-4 = 0 = Less than daily  1 = Daily  9 = Missing |
|  | Alcohol habits | Alcohol consumption | 1 = More than twice a week  2 = Up to twice a week  3 = Once a week  4 = Once to twice a month  5 = Never  9 = Wrong answer | 4-5 = 0 = A couple of times monthly/never  1-3 = 1 = One to several times weekly  9 = Missing |
|  | Self-reported weight and height | Body mass index (BMI) | Calculated as weight in kilograms divided by height in meters squared | 1 = Underweight (<18.5)  2 = Normal weight (18.5–24.9)  3 = Overweight (25–29.9)  4 = Obese (>30 )  9= No answer |
| *Self-perceived oral and general health* | Are you satisfied with your oral health? | **Perceived oral health** | 1 = Yes, very satisfied  2 = Yes, largely satisfied  3 = No, not very satisfied  4 = No, absolutely not satisfied  9 = Wrong answer | 1-2 = 0 = Very/Largely satisfied 3-4 = 1 = Not very/Absolutely not satisfied  9 = Missing |
|  | Do you consider yourself healthy? | **Perceived general health** | 1 = Yes, absolutely  2 = Yes, largely  3 = No, not very  4 = No, absolutely not  5 = Have no idea | 1-2 = 0 = Healthy  3-4 = 1 = Not healthy  5=2= Have no opinion |
|  | How many of your own teeth do you have? | Remaining teeth | 1 = All remaining teeth  2 = Missing a single tooth  3 = Missing many teeth  4 = Almost no teeth less  5 = Edentulous | 1-2 = 0 = All remaining/Missing a single tooth 3-5 = 1 = Missing many/Almost no remaining |
|  | When did you have toothache latest? | Toothache | 1 = During the last three months  2 = During last year  3 = More than one year ago  4 = Never had  5 = Do not remember | 4-5 = 0 = More than one year ago/never had  1-3 = 1 = During the last three months/ under last year |
|  | Can you chew all types of food? | Chewing | 1 = Very good  2 = Relatively good  3 = Not so good  4 = Bad  9 = Wrong answer | 1-2 = 0 = Very /rather good  3-4 = 1 = Less good/ /bad  9 = Missing |
|  | Do you feel dry mouth during the day? | Dry mouth during the day | 1 = Yes, often  2 = Yes, sometimes  3 = No, rarely  4 = No, never  9 = Wrong answer | 1-3 = 0 = Seldom, sometimes or often  4 = 1 = Never  9 = Missing |
|  | Do you feel dry mouth at night? | Dry mouth at night | 1 = Yes, often  2 = Yes, sometimes  3 = No, rarely  4 = No, never  9 = Wrong answer | 1-3 = 0 = Seldom, sometimes or often  4 = 1 = Never  9 = Missing |
| *Dependency* | Do you need daily help with… | Dependency | 1 = cleaning  2 = cooking  3 = buying food  4 = personal hygiene  Each category has been recoded to a binary variable:  0 = No  1 = Yes | Dependency = 1 if at least one of any options was 1, otherwise = 0 |

**Supplementary Table S2**. Response rate per survey wave.

|  | Survey year | Age, yrs | Female (%) responders | Male (%) responders | Total (%) responders | Total (% of response at baseline) |
| --- | --- | --- | --- | --- | --- | --- |
| **1992 cohort** | 1992 | 50 | 3,181 (50.2) | 3,160 (49.8) | 6,341 (71.4) |  |
|  | 1997 | 55 | 3,321 (51.0) | 3,186 (49.0) | 6,507 (74.3) |  |
|  | 2002 | 60 | 3,261 (51.2) | 3,107 (48.8) | 6,368 (75.0) |  |
|  | 2007 | 65 | 3,077 (50.7) | 2,998 (49.4) | 6,075 (73.1) |  |
|  | 2012 | 70 | 2,891 (50.8) | 2,800 (49.2) | 5,691 (72.2) |  |
|  | 2017 | 75 | 2,641 (51.9) | 2,445 (48.1) | 5,086 (70.7) |  |
|  | *Cohort by survey year* | | | | | |
|  | 92/97/02/07/12/17 |  | 1,633 (53.4) | 1,425 (46.6) |  | 3,058 (48.2) |
|  |  |  |  |  |  |  |
| **2007 cohort** | 2007 | 75 | 1,987 (53.2) | 1,745 (46.8) | 3,732 (71.9) |  |
|  | 2012 | 80 | 1,594 (54.6) | 1,325 (45.4) | 2,919 (66.8) |  |
|  | 2017 | 85 | 1,131 (56.2) | 883 (43.8) | 2,014 (61.6) |  |
|  | *Cohort by survey year* | | | | | |
|  | 07/12/17 |  | 859 (54.2) | 727 (45.8) |  | 1,586 (42.5) |

**Supplementary Table S3**. Logistic regression results for the association between nonresponse to baseline surveys and baseline socio-demographic variables obtained from National Registers in men and women–complete-case analysis.

|  | **1992 cohort (born in 1942)** | |  | **2007 cohort (born in 1932)** | |
| --- | --- | --- | --- | --- | --- |
|  | **Men**  **(N=4,482)** | **Women**  **(N=4,320)** |  | **Men**  **(N=2,772)** | **Women**  **(N=2,298)** |
| **Sociodemographic characteristics** | **aOR (95% CI)** | **aOR (95% CI)** |  | **aOR (95% CI)** | **aOR (95% CI)** |
| **Country of birth** |  |  |  |  |  |
| Sweden | Ref. | Ref. |  | Ref. | Ref. |
| Foreign country | 1.40 [1.12,1.77] | 1.48 [1.14,1.91] |  | 1.73 [1.26,2.37] | 2.01 [1.53,2.65] |
| **Residential area by degree of urbanization** |  |  |  |  |  |
| Degree 1(cities) | Ref. | Ref. |  | Ref. | Ref. |
| Degree 2 (towns & suburbs) | 1.01 [0.86,1.19] | 1.03 [0.87,1.23] |  | 0.93 [0.73,1.18] | 0.92 [0.74,1.13] |
| Degree 3 (rural areas) | 1.02 [0.86,1.20] | 1.05 [0.88,1.25] |  | 1.11 [0.87,1.41] | 1.04 [0.84,1.29] |
| **Education** |  |  |  |  |  |
| Compulsory | Ref. | Ref. |  | Ref. | Ref. |
| Secondary | 0.73 [0.63,0.84] | 0.77 [0.66,0.90] |  | 0.73 [0.59,0.91] | 0.63 [0.52,0.76] |
| Post-secondary | 0.57 [0.47,0.70] | 0.57 [0.46,0.71] |  | 0.58 [0.41,0.82] | 0.43 [0.31,0.60] |
| **Marital status** |  |  |  |  |  |
| Unmarried | 1.61 [1.34,1.93] | 1.35 [1.07,1.71] |  | 2.29 [1.64,3.19] | 2.22 [1.50,3.28] |
| Married/cohabiting | Ref. | Ref. |  | Ref. | Ref. |
| Divorced/separated | 1.46 [1.22,1.74] | 1.74 [1.46,2.08] |  | 1.71 [1.30,2.26] | 1.62 [1.24,2.12] |
| Widower | 0.91 [0.45,1.87] | 1.85 [1.24,2.76] |  | 1.95 [1.43,2.65] | 1.44 [1.17,1.78] |
| **Tertiles of disposable income** |  |  |  |  |  |
| 1 (low) | Ref. | Ref. |  | Ref. | Ref. |
| 2 (medium) | 0.62 [0.52,0.75] | 0.72 [0.62,0.85] |  | 0.66 [0.50,0.87] | 0.83 [0.68,1.03] |
| 3 (high) | 0.53 [0.44,0.63] | 0.69 [0.55,0.86] |  | 0.45 [0.34,0.60] | 0.67 [0.51,0.87] |
| **County** |  |  |  |  |  |
| Östergötlands län | Ref. | Ref. |  | Ref. | Ref. |
| Örebro län | 0.99 [0.87,1.13] | 1.00 [0.87,1.15] |  | 1.05 [0.86,1.29] | 1.04 [0.87,1.24] |

Abbreviations: aOR = adjusted odds ratio; CI = Confidence Interval

**Supplementary Table S4a.** Factors associated with dropout. Results from logistic regression analysis using the observed data to assess the extent to which variables at baseline survey in 1992 predict dropout from subsequent waves in individuals born in 1942 who participated in baseline survey (people who died or emigrated are excluded from these analyses).

|  | **1997 survey** | **2002 survey** | **2007 survey** | **2012 survey** | **2017 survey** |
| --- | --- | --- | --- | --- | --- |
|  | **N_R_=5,247**  **N_NR_ = 674**  **N_A_ =5,921** | **N_R_=4,991**  **N_NR_=657**  **N_A_ =5,648** | **N_R_=4,605**  **N_NR_=801**  **N_A_ =5,406** | **N_R_=4,225**  **N_NR_=810**  **N_A_ =5,035** | **N_R_=3,766**  **N_NR_=865**  **N_A_ =4,631** |
|  | **aOR (95% CI)** | **aOR (95% CI)** | **aOR (95% CI)** | **aOR (95% CI)** | **aOR (95% CI)** |
| ***Sociodemographic characteristics from National Registers*** |  |  |  |  |  |
| **Male sex** | 1.55 [1.28,1.87] | 1.64 [1.35,1.99] | 1.25 [1.04,1.49] | 1.14 [0.95,1.37] | 1.17 [0.98,1.40] |
| **Country of birth** |  |  |  |  |  |
| Sweden | Ref. | Ref. | Ref. | Ref. | Ref. |
| Foreign country | 1.52 [1.13,2.05] | 1.84 [1.37,2.46] | 2.00 [1.53,2.63] | 1.85 [1.39,2.45] | 1.64 [1.23,2.19] |
| **Residential area by degree of urbanization** |  |  |  |  |  |
| Degree 1(cities) | Ref. | Ref. | Ref. | Ref. | Ref. |
| Degree 2 (towns & suburbs) | 1.07 [0.88,1.31] | 1.05 [0.86,1.29] | 1.05 [0.87,1.26] | 1.03 [0.85,1.25] | 1.24 [1.03,1.49] |
| Degree 3 (rural areas) | 1.07 [0.87,1.32] | 1.02 [0.83,1.27] | 0.92 [0.75,1.12] | 0.95 [0.78,1.16] | 1.10 [0.90,1.33] |
| **Education** |  |  |  |  |  |
| Compulsory | Ref. | Ref. | Ref. | Ref. | Ref. |
| Secondary | 0.90 [0.75,1.08] | 0.80 [0.66,0.96] | 0.78 [0.66,0.93] | 0.71 [0.60,0.85] | 0.71 [0.60,0.84] |
| Post-secondary | 0.82 [0.64,1.05] | 0.69 [0.54,0.90] | 0.63 [0.50,0.80] | 0.59 [0.46,0.74] | 0.54 [0.42,0.68] |
| **Marital status** |  |  |  |  |  |
| Unmarried | 1.36 [1.06,1.74] | 1.53 [1.19,1.96] | 1.71 [1.36,2.15] | 1.78 [1.41,2.25] | 1.79 [1.41,2.26] |
| Married/cohabiting | Ref. | Ref. | Ref. | Ref. | Ref. |
| Divorced/separated | 1.16 [0.92,1.46] | 1.62 [1.30,2.02] | 1.17 [0.93,1.45] | 1.44 [1.15,1.79] | 1.40 [1.13,1.74] |
| Widower | 1.18 [0.63,2.20] | 0.99 [0.49,1.99] | 1.67 [0.99,2.81] | 1.96 [1.16,3.31] | 1.37 [0.78,2.38] |
| **Tertiles of disposable income** |  |  |  |  |  |
| 1 (low) | Ref. | Ref. | Ref. | Ref. | Ref. |
| 2 (medium) | 0.86 [0.70,1.05] | 0.79 [0.64,0.98] | 0.82 [0.68,1.00] | 0.82 [0.68,0.99] | 0.83 [0.69,1.01] |
| 3 (high ) | 0.76 [0.60,0.97] | 0.65 [0.51,0.83] | 0.74 [0.59,0.93] | 0.62 [0.49,0.78] | 0.69 [0.55,0.87] |
| **County** |  |  |  |  |  |
| Östergötlands län | Ref. | Ref. | Ref. | Ref. | Ref. |
| Örebro län | 1.12 [0.95,1.33] | 1.14 [0.97,1.36] | 1.05 [0.89,1.22] | 0.95 [0.81,1.12] | 1.03 [0.88,1.21] |
| ***Variables from baseline survey in 1992*** |  |  |  |  |  |
| **Smoking** |  |  |  |  |  |
| Less than daily | Ref. | Ref. | Ref. | Ref. | Ref. |
| Daily | 1.69 [1.42,2.00] | 1.53 [1.28,1.83] | 1.53 [1.30,1.80] | 1.70 [1.44,2.00] | 1.45 [1.23,1.72] |
| **Brushing** |  |  |  |  |  |
| Twice a day or more | Ref. | Ref. | Ref. | Ref. | Ref. |
| Less than twice a day | 1.23 [1.00,1.51] | 1.07 [0.86,1.33] | 1.11 [0.90,1.36] | 1.15 [0.93,1.42] | 1.15 [0.93,1.41] |
| **Perceived oral health** |  |  |  |  |  |
| Very/largely satisfied | Ref. | Ref. | Ref. | Ref. | Ref. |
| Not very/absolutely not satisfied | 1.57 [1.30,1.91] | 1.38 [1.13,1.68] | 1.27 [1.05,1.53] | 1.48 [1.23,1.78] | 1.27 [1.05,1.54] |
| **Perceived general health** |  |  |  |  |  |
| Healthy | Ref. | Ref. | Ref. | Ref. | Ref. |
| Not healthy | 1.25 [0.97,1.59] | 1.41 [1.10,1.80] | 1.23 [0.97,1.56] | 1.61 [1.28,2.03] | 1.71 [1.35,2.16] |
| Have no opinion | 1.55 [0.61,3.90] | 1.70 [0.66,4.36] | 1.54 [0.60,3.98] | 0.79 [0.22,2.81] | 1.08 [0.34,3.41] |

Note: Observations with missing baseline values (2.6%) are excluded from the analysis.

Abbreviations: aOR = adjusted odds ratio; CI = Confidence Interval; N_R_ = number of respondents, N_NR_= number of non-respondents, N_A_ = number in the adjusted model.

**Supplementary Table S4b**. Factors associated with dropout. Results from logistic regression using the observed data to assess the extent to which variables at baseline survey in 2007 predict dropout from subsequent waves in individuals born in 1932 who participated in baseline survey (people who died or emigrated are excluded from these analyses).

|  | **2012 survey** | **2017 survey** |
| --- | --- | --- |
|  | **N_R_=2,279**  **N_NR_=507**  **N_A_ =2,786** | **N_R_=1,531**  **N_NR_=601**  **N_A_ =** **2,132** |
|  | **aOR (95% CI)** | **aOR (95% CI)** |
| ***Sociodemographic characteristics from National Registers*** |  |  |
| **Male sex** | 1.17 [0.91,1.49] | 0.78 [0.61,1.00] |
| **Country of birth** |  |  |
| Sweden | Ref. | Ref. |
| Foreign country | 1.31 [0.91,1.89] | 1.23 [0.84,1.79] |
| **Residential area by degree of urbanization** |  |  |
| Degree 1(cities) | Ref. | Ref. |
| Degree 2 (towns & suburbs) | 0.74 [0.58,0.95] | 0.95 [0.75,1.21] |
| Degree 3 (rural areas) | 0.93 [0.72,1.19] | 1.01 [0.79,1.29] |
| **Education** |  |  |
| Compulsory | Ref. | Ref. |
| Secondary | 0.80 [0.64,0.99] | 0.83 [0.67,1.02] |
| Post-secondary | 0.65 [0.46,0.91] | 0.50 [0.36,0.69] |
| **Marital status** |  |  |
| Unmarried | 1.41 [0.90,2.22] | 1.04 [0.64,1.70] |
| Married/cohabiting | Ref. | Ref. |
| Divorced/separated | 1.08 [0.78,1.50] | 1.33 [0.98,1.82] |
| Widower | 1.60 [1.23,2.08] | 1.22 [0.94,1.60] |
| **Tertiles of disposable income** |  |  |
| 1 ( low) | Ref. | Ref. |
| 2 (medium) | 0.69 [0.53,0.89] | 0.72 [0.55,0.94] |
| 3 ( high ) | 0.52 [0.39,0.70] | 0.74 [0.56,0.99] |
| **County** |  |  |
| Östergötlands län | Ref. | Ref. |
| Örebro län | 1.11 [0.90,1.36] | 1.08 [0.88,1.31] |
| ***Variables from baseline survey in 2007*** |  |  |
| **Smoking** |  |  |
| Less than daily | Ref. | Ref. |
| Daily | 1.73 [1.20,2.50] | 1.68 [1.12,2.51] |
| **Brushing** |  |  |
| Twice a day or more | Ref. | Ref. |
| Less than twice a day | 1.23 [0.95,1.59] | 1.21 [0.93,1.57] |
| **Perceived oral health** |  |  |
| Very satisfied/largely satisfied | Ref. | Ref. |
| Not very/absolutely not satisfied | 1.26 [1.00,1.61] | 1.03 [0.80,1.33] |
| **Perceived general health** |  |  |
| Healthy | Ref. | Ref. |
| Not healthy | 1.36 [1.09,1.70] | 1.42 [1.13,1.78] |
| Have no opinion | 1.76 [0.75,4.13] | 1.62 [0.62,4.27] |

Note: Observations with missing baseline values (12.7% and 12.1% of the eligible in 2012 and 2017, respectively) are excluded from the analysis.

Abbreviations: aOR = adjusted odds ratio; CI = Confidence Interval; N_R_ = number of respondents, N_NR_= number of non-respondents, N_A_ = number in the adjusted model.

**Supplementary Table S5**. Factors associated with attrition. Results from logistic regression using the observed data to assess the extent to which variables at previous survey predict dropout from subsequent wave (people who died or emigrated are excluded from these analyses).

|  | **1992 cohort** | | | | **2007 cohort** |
| --- | --- | --- | --- | --- | --- |
|  | **2002 survey** | **2007 survey** | **2012 survey** | **2017 survey** | **2017 survey** |
|  | **N_R_=5079**  **N_NR_=696**  **N_A_ =5,775** | **N_R_=4679**  **N_NR_=831**  **N_A_ =5,510** | **N_R_=4308**  **N_NR_=838**  **N_A_ =5,146** | **N_R_=3,839**  **N_NR_=882**  **N_A_ =4,721** | **N_R_=1,722**  **N_NR_=681**  **N_A_ =2,403** |
|  | **aOR (95% CI)** | **aOR (95% CI)** | **aOR (95% CI)** | **aOR (95% CI)** | **aOR (95% CI)** |
| **Sociodemographic characteristics from National Registers for the previous survey year** |  |  |  |  |  |
| **Marital status** |  |  |  |  |  |
| Unmarried | 1.41 [1.08,1.86] | 1.60 [1.23,2.09] | 1.46 [1.11,1.93] | 1.68 [1.26,2.25] | 0.97 [0.60,1.58] |
| Married/cohabiting | Ref. | Ref. | Ref. | Ref. | Ref. |
| Divorced/separated | 1.47 [1.18,1.84] | 1.16 [0.94,1.45] | 1.37 [1.10,1.70] | 1.45 [1.16,1.81] | 1.52 [1.12,2.06] |
| Widower | 1.13 [0.65,1.99] | 1.40 [0.95,2.06] | 1.31 [0.94,1.81] | 1.34 [1.02,1.78] | 1.31 [1.02,1.69] |
| **Tertiles of disposable income** |  |  |  |  |  |
| 1 (low) | Ref. | Ref. | Ref. | Ref. | Ref. |
| 2 (medium) | 0.68 [0.55,0.84] | 0.82 [0.67,1.01] | 0.76 [0.62,0.93] | 0.80 [0.65,0.99] | 0.92 [0.71,1.19] |
| 3 (high) | 0.60 [0.46,0.76] | 0.63 [0.49,0.80] | 0.59 [0.47,0.75] | 0.72 [0.57,0.92] | 0.87 [0.66,1.16] |
| **Information from previous survey (t-1)** |  |  |  |  |  |
| **Smoking** |  |  |  |  |  |
| Less than daily | Ref. | Ref. | Ref. | Ref. | Ref. |
| Daily | 1.70 [1.34,2.16] | 1.93 [1.53,2.42] | 2.05 [1.59,2.66] | 1.18 [0.82,1.70] | 1.53 [0.91,2.57] |
| **Brushing** |  |  |  |  |  |
| Twice a day or more | Ref. | Ref. | Ref. | Ref. | Ref. |
| Less than twice a day | 1.12 [0.86,1.47] | 1.32 [1.03,1.70] | 1.27 [0.98,1.66] | 1.42 [1.09,1.86] | 1.16 [0.85,1.58] |
| **Perceived oral health** |  |  |  |  |  |
| Very satisfied/largely satisfied | Ref. | Ref. | Ref. | Ref. | Ref. |
| Not very/absolutely not satisfied | 1.10 [0.85,1.44] | 1.14 [0.91,1.44] | 1.24 [0.98,1.57] | 0.90 [0.68,1.17] | 0.88 [0.65,1.19] |
| **Perceived general health** |  |  |  |  |  |
| Healthy | Ref. | Ref. | Ref. | Ref. | Ref. |
| Not healthy | 1.21 [0.91,1.60] | 1.10 [0.87,1.39] | 1.44 [1.14,1.83] | 1.71 [1.34,2.20] | 1.34 [1.05,1.71] |
| Have no opinion | 2.16 [0.84,5.52  ] | 1.41 [0.47,4.23] | 1.42 [0.53,3.78] | 0.73 [0.21,2.46] | 1.28 [0.47,3.48] |

Note: Estimates for time-invariant variables (sex, country of birth), as well as time-varying variables that were highly correlated (correlation coefficient >.7) with baseline counterparts (such as urbanization degree of residential area, county, education) are not shown although they are included in the adjusted model. Observations with missing values from register-based data (0.4-0.9%) are excluded from the analyses. Observations with missing values from follow-up surveys (for smoking, brushing, oral and general health) are included using missing value indicator as proportion of missing values ranged from 12.1% to 30.0%.

Abbreviations: aOR = adjusted odds ratio; CI = Confidence Interval; N_R_ = number of respondents, N_NR_= number of non-respondents, N_A_ = number in the adjusted model.
